# COVID-19 Knowledge and Prevention Behaviors in Rural Zambia: A Qualitative Application of the Information-Motivation-Behavioral Skills Model

**DOI:** 10.4269/ajtmh.22-0604

**Published:** 2023-05-30

**Authors:** Jeanette L. Kaiser, Davidson H. Hamer, Allison Juntunen, Thandiwe Ngoma, Günther Fink, Jessica Schueler, Peter C. Rockers, Godfrey Biemba, Nancy A. Scott

**Affiliations:** ^1^ Department of Global Health, Boston University School of Public Health, Boston, Massachusetts;; ^2^ Section of Infectious Diseases, Boston University School of Medicine, Boston, Massachusetts;; ^3^ National Emerging Infectious Diseases Laboratory, Boston University, Boston, Massachusetts;; ^4^ Center for Emerging Infectious Diseases Policy and Research, Boston University, Boston, Massachusetts;; ^5^ Right to Care Zambia, Lusaka, Zambia;; ^6^ Swiss Tropical and Public Health Institute and University of Basel, Basel, Switzerland;; ^7^ National Health Research Authority, Lusaka, Zambia

## Abstract

In early 2020, the Zambian Ministry of Health instituted prevention guidelines to limit spread of COVID-19. We assessed community knowledge, motivations, behavioral skills, and perceived community adherence to prevention behaviors (i.e., hand hygiene, mask wearing, social distancing, and limiting gatherings). Within a cluster-randomized controlled trial in four rural districts, in November 2020 and May 2021, we conducted in-depth interviews with health center staff (*N* = 19) and community-based volunteers (*N* = 34) and focus group discussions with community members (*N* = 281). A content analysis was conducted in Nvivo v12. Data were interpreted using the Information-Motivation-Behavioral Skills Model. Generally, respondents showed good knowledge of COVID-19 symptoms, spread, and high-risk activities, with some gaps. Prevention behavior performance was driven by personal and social factors. Respondents described institutional settings (e.g., clinics and church) having higher levels of perceived adherence due to stronger enforcement measures and clear leadership. Conversely, informal community settings (e.g., weddings, funerals, football matches) lacked similar social and leadership expectations for adherence and had lower perceived levels of adherence. These settings often involved higher emotions (excitement or grief), and many involved alcohol use, resulting in community members “forgetting” guidelines. Doubt about disease existence or need for precautions persisted among some community members and drove non-adherence more generally. Although COVID-19 information successfully penetrated these very remote rural communities, more targeted messaging may address persistent COVID-19 doubt and misinformation. Engaging local leaders in religious, civic, and traditional leadership positions could improve community behaviors without adding additional monitoring duties on an already overburdened, resource-limited health system.

## INTRODUCTION

Zambia, a lower-middle income country in sub-Saharan Africa, with a population of approximately 18 million people, has been affected by the 2019 coronavirus disease (COVID-19).^1^^,^^2^ Caused by the SARS-CoV-2 virus, COVID-19 is an acute respiratory illness. As of July 2022, there had been over 571 million confirmed cases worldwide and over 6.3 million deaths.^3^ COVID-19 was first confirmed in Zambia on March 18, 2020.^4^^–^^6^ To date, Zambia has experience four infections peaks, each progressively higher than the last. Peaks were seen in August 2020, December 2020/January 2021 (driven by the beta variant), June/July 2021 (driven by the delta variant), and December 2021/January 2022 (driven by the omicron variant).^7^^,^^8^ As of August 2022, there had been over 329,000 confirmed cases and over 4,000 documented deaths from COVID-19 in the country.^8^ The epidemic was initially concentrated in urban areas, particularly in the capital city of Lusaka, but as detected cases increased, transmission became more widespread in rural areas.^9^

It is now known that large crowds, particularly indoors with poor ventilation and without face coverings (masks), drive rapid transmission.^10^ One systematic review confirmed that, in addition to hand hygiene, the best strategies for decreasing transmission risk include being distant (at least 2 m) from infected individuals and wearing face masks and eye coverings.^11^ Mask wearing has been shown to limit the risk of transmission by using electrostatic filtration to trap particles from crossing the barrier; although N95 masks are ideal, even multi-layered cloth masks effectively block particles and prevent transmission.^12^^,^^13^

In March 2020, in addition to other lockdown measures, the Zambian Ministry of Health (MoH) issued guidelines for multiple non-pharmaceutical interventions (NPIs) to slow the transmission of COVID-19, including requiring face masks and social distancing of at least 1 m in public settings and the use of alcohol-based hand sanitizers or handwashing with soap and water.^6^^,^^14^ The MoH guidelines limited attendance at gatherings to 10 or 50 persons, depending on severity of the country epidemic at the time. To generate awareness about the disease and the prevention guidelines, the MoH developed a series of health information, education, and communication (IEC) materials, in English and in the seven main local languages, about COVID-19 in early 2020. Posters and brochures were distributed to health centers and communities around the country via the provincial and district health offices. Information was also routinely disseminated through radio and television broadcasts and social media.

Although the personal and social benefits of following NPI guidelines for containing the spread of COVID-19 have been demonstrated,^11^^,^^15^^–^^18^ the behavior changes required have proved challenging to attain and maintain in many settings. Health behaviors are difficult to modify, particularly in the absence of tangible, immediate harms. Countless theories and models have been developed to explain the external and internal factors and relationships that influence individual behavior change in the short or long term.^19^^,^^20^ Health behavior theories have been developed and applied to a vast range of health-related actions, from eating a nutritious diet, to correct and consistent condom use during sexual intercourse, to HIV or TB medication adherence.^20^^,^^21^ One simple model to understand the psychological underpinnings of health behaviors is the Information-Motivation-Behavioral Skills Model (IMB Model), first developed by Fisher and Fisher in the early 1990s for HIV risk and prevention behaviors.^22^ This model theorizes that information, motivation, and the skills to perform behavior are prerequisites for the behavior actions needed to affect health outcomes. The model has been widely applied to disease and injury prevention behaviors, disease screening and diagnosis behaviors, and medication adherence.^22^ Assessed through multiple quantitative studies, the constructs of information, motivation, and behavior skills have been found to be statistically linked with health behavior performance, either independently or through association with one of the other constructs, depending on the complexity and novelty of the assessed behavior(s).^22^

Applying the IMB Model to behaviors associated with COVID-19 transmission prevention, we assessed the knowledge, motivations, skills, and perceived community-level adherence to MoH guidelines in rural Zambian communities using qualitative data collected in late 2020 and mid-2021.

## MATERIALS AND METHODS

### Study setting.

This qualitative study was conducted within a large cluster-randomized controlled trial that aimed to assess the impact of two early childhood development interventions in Choma, Kalomo, and Pemba Districts of Southern Province and Nyimba District of Eastern Province.^23^ These districts are predominantly rural, with low population densities outside of small urban areas that serve as the centers of district administration and commerce. Rural communities in these districts are generally subsistence farmers or work in the agricultural sector and have limited access to electricity, piped water, or improved sanitation methods.^24^^,^^25^

Rural health centers in Zambia are public facilities that provide the surrounding populations (known as populations living in their catchment areas) with a wide range of primary health care services, including preventive and minor curative services. A varied cadre of community-based volunteers (CBVs) have been trained to provide health promotion services in maternal, newborn, and child health; water, sanitation, and hygiene; and/or infectious disease control over time.^26^ CBVs routinely interact with some of the most remote communities within their health facility catchment areas. For the health centers included in this study, the median distance to the furthest village in their catchment area was 17.3 km (interquartile range: 14.2–21.6).

### Theoretical framework.

**Figure 1.**
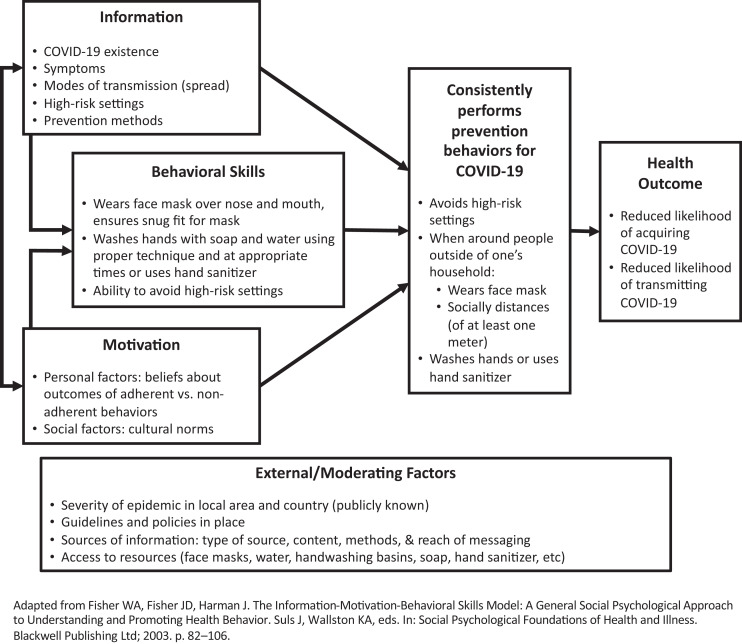
Information-motivation-behavioral skills model adapted to COVID-19 transmission prevention behaviors.

We interpret and present our findings using adapted domains of the IMB Model: information, motivators, behavioral skills, and finally behaviors (Figure 1).^22^ The ultimate behavior is interpreted as perceptions of community adherence to COVID-19 guideline prevention methods, including avoiding high-risk settings such as large crowds, wearing face masks, social distancing of at least 1 m, and frequent hand washing with soap and water or use of alcohol-based hand sanitizers. We posit that having correct information available on the existence of COVID-19, its symptoms, modes of transmission, high-risk settings for transmission, and prevention methods influences eventual application of prevention behaviors. Motivations for performing the behavior can exist at the personal (e.g., beliefs, fears, etc.) or social (e.g., example of leadership, peer pressure, enforcement, social repercussions, etc.) levels. Specific factors motivating or demotivating remote Zambian communities to adhere to the COVID-19 prevention behaviors are discussed in the findings below. We interpret behavioral skills as the knowledge and ability to perform prevention behaviors correctly (e.g., proper handwashing or face mask wearing technique) and at the correct times.

A number of external and moderating factors likely influence information availability, motivation to perform the behaviors, and behavioral skills. We believe these include the severity of the epidemic locally and nationally; established guidelines and policies; content, methods, and reach of official IEC efforts; and the content, amount, and reach of media coverage, including social media and news sources. Additionally, especially in resource-limited settings, access to the resources needed to perform the behaviors, including masks, water, handwashing basins, soap, and hand sanitizer can be economically or geographically inaccessible.

### Study design.

We conducted two cross-sectional qualitative rounds of data collection on community perceptions, knowledge, and behaviors around COVID-19 and the associated government guidelines. In the catchment areas of 10 rural health centers, we conducted in-depth interviews (IDIs) with health center staff and CBVs and focus group discussion (FGDs) with community members.

### Sampling.

One health center staff member was purposively sampled from each rural health center based on availability during data collection (*N* = 19). A total of 34 CBVs were purposively selected and interviewed. A total of 43 FGDs, each with six to eight participants (*N* = 281), were conducted in the surrounding communities. Participants were purposively selected by CBVs from the relevant areas if they had a young child in their household and had participated in one of the ECD interventions implemented under the larger evaluation.

### Data collection methods.

The semi-structured IDI and FGD instruments included questions on what the respondents know about COVID-19 in general, its symptoms, spread, and what activities could make the disease more likely to spread, discussed in the results as “high-risk activities.” The instruments included a question on respondents’ sources of information about COVID-19 in the surrounding communities and multiple questions on knowledge of current COVID-19 prevention guidelines. Lastly, respondents were asked about their perceptions of guideline enforcement of prevention guidelines, examples and reasons for adherence to guidelines, and examples and reasons for non-adherence. Probes were used to elicit more information.

The qualitative instruments were translated into the local languages of Chitonga and Chinyanja for use in the Southern and Eastern Provinces, respectively. Project staff trained a team of local data collectors on research ethics, on principles of qualitative data collection, and on the data collection instruments. Round 1 of data collection occurred over 1 month in November 2020. Round 2 followed and lasted 1 month during April/May 2021. Prevention measures were followed to limit the possibility of transmission of COVID-19 during data collection activities, including face mask use, social distancing, and use of alcohol-based hand sanitizers.

Demographic data were collected from each respondent using paper forms. Data were extracted at a later time using SurveyCTO® Collect Software (Dobility, Inc, Cambridge, MA) on encrypted tablets.

### Ethics.

Ethical approvals for the overarching evaluation and the protocol amendments made to include collection of COVID-19–related perception and behavior data were obtained from the Boston University Medical Campus Institutional Review Board and the University of Zambia Biomedical Research Ethics Committee. The overarching evaluation was approved by the National Health Research Authority; the MoH at the national, provincial, and district levels; and traditional chiefs overseeing the local areas. Written informed consent was obtained from each participant in their language of choice and documented with a signature or thumb print. IDIs and FGDs were audio recorded with consent from each participant. CBVs and community members received a small token of appreciation for their time. Health center staff did not receive any tokens in accordance with MoH guidelines.

### Data analysis.

The audio recordings were concurrently translated into English and transcribed into Microsoft Word by individuals fluent in English and one or both local languages. A mixed inductive-deductive approach was used for coding. An initial codebook was created a priori based on the questions in the instruments. During the coding process, additional sub-codes were added as themes emerged. All coding and analysis was conducted in Nvivo v12 (QSR International, Doncaster, Australia). Three study personnel conducted the coding. At the beginning of each round of analysis, personnel each coded the same interviews to measure inter-rater reliability. We used the Nvivo Coding Comparison query and considered the resulting kappa coefficients. We ensured that the coefficients were over 0.75 each time, per Nvivo’s recommendation, and that coding was as similar as possible among individuals.^27^ Where there were differences in the alignment, the team discussed discrepancies and came to an agreement. Content analysis was conducted using matrix queries to compare responses for each code by respondent type to identify themes. Themes related to knowledge of aspects of COVID-19 disease and prevention guidelines are presented as the “Information” construct of the IMB Model. Themes of enforcement of guidelines and reasons for guideline adherence or non-adherence are presented as the “Motivation” construct. Themes related to knowledge of proper methods of adherence and settings where adherence is most critical are considered “‘Behavioral skills.” Dominant themes were summarized and included in the results. Although we analyzed each round of data collection separately, results presented below apply to both rounds of data collection unless otherwise specified; we highlight any key differences between rounds.

Although findings from this analysis could not be validated by the participants, they were triangulated with previously published quantitative data from the same communities on attendance at community gatherings and adherence to guidelines.^28^ Our qualitative findings were also verified at a local stakeholders’ meeting. Findings are reported according to the Standards for Reporting Qualitative Research Checklist.^29^

Demographic data were cleaned and analyzed in SAS v9.4 (SAS Institute Inc., Cary, NC). Descriptive characteristics are presented by respondent type. Means and standard deviations were calculated for age highest grade completed. Proportions were calculated for respondent sex, marital status, and clinical position of health center staff. We only collected marital status for FGD respondents. We first present the demographic characteristics of the respondents and then the qualitative results by theme.

## RESULTS

### Demographic characteristics of respondents.

A total of 334 individuals participated in this qualitative study; 19 health center staff and 34 CBVs participated in IDIs, and 281 community members participated in FGDs (Table 1). The majority of CBVs and community members were female. CBVs were generally older than the other respondents, and health center staff had completed more years of education. Nearly half of health center staff were nurses and/or midwives (47.4%), with the remaining being environmental health technologists (EHTs; 36.8%) or clinical officers (15.8%).

**Table 1:** Demographic characteristics by respondent type (*N* = 334)

Characteristic	Health center staff (*N* = 19)	Community-based volunteers (*N* = 34)	Community members (*N* = 281)
Female, *n* (%)	7 (36.8)	19 (55.9)	201 (71.5)
Age, mean (SD)	32.2 (8.0)	45.9 (11.0)	31.2 (8.9)
Highest grade completed, mean (SD)	13.0 (0)	9.9 (2.0)	7.6 (3.0)
Married/cohabiting, *n* (%)	–	–	248 (88.3)
Data collection round, *n* (%)			
Round 1: November 2020	10 (52.6)	14 (41.2)	117 (41.6)
Round 2: April/May 2021	9 (47.4)	20 (58.8)	164 (58.4)

### Information: knowledge about COVID-19.

#### General knowledge and symptoms.

In their initial explanations, when asked what they know about COVID-19, health center staff described COVID-19 scientifically, discussing it as a disease caused by a virus or infection, noting that it affects the respiratory system and is transmissible, with multiple respondents specifically explaining that transmission occurs through respiratory droplets. Many noted when and where the virus originated and/or that it has spread worldwide. CBVs and community members provided a simpler description of COVID-19, calling it a disease or virus, noting that it is dangerous and that it can be deadly but that it is preventable, with many mentioning its transmissibility and modes of transmission. Many respondents immediately began describing prevention methods outlined in the MoH guidelines. Illustrative quotes are included below:

*“COVID-19 is a respiratory tract infection which is transmitted or contracted through droplets, handshakes and if you are found in a crowded area you can easily get it.” – Health center staff, female, Pemba district, round 1*

*“[People] say COVID-19 is a vicious disease which has killed so many people. So, we are told on how we are supposed to protect ourselves…” – Community member, female, Kalomo district, round 2*

Coughing was widely discussed as a main symptom of COVID-19 by all respondent groups, along with three other main symptoms: fever, difficulty breathing, and nasal symptoms. Some respondents also discussed fatigue and body weakness, headache, sore throat, body or muscle pains, and chest pain as symptoms of COVID-19. Loss of taste and/or smell was rarely mentioned by any respondent group. A few illustrative quotes are included below:

*“If someone contracted COVID-19, they will have fever, cough, sneezing, sore throat and at times general body pains as well.” – Health center staff, male, Nyimba district, round 1*

*“According to what we were taught, if you experience difficulty in breathing, coughing… have flu and feeling tiredness all the time. These are the signs they showed that this could be coronavirus.” – Community member, male, Kalomo district, round 2*

#### Modes of transmission: spread.

Coughing was widely discussed as the main mode of transmission of the disease. Health center staff specified that the mechanism of transmission is through respiratory or saliva droplets, with some describing the virus as airborne (Table 2). Many community members described coughing, sneezing, talking, breathing, and “exchanging air” as drivers of disease spread. Additionally, respondents focused on people’s actions related to not following prevention methods, which could result in spreading the disease: 1) being in crowds without proper social distancing (described as “sitting close” or hugging), where droplets could fall on someone sitting nearby; 2) not wearing a face mask that would prevent transmission of fluids from an infected individual’s cough to an uninfected one; and 3) touching one’s own face without washing their hands after holding hands with someone else, shaking hands, or touching a potentially contaminated surface.

**Table 2:** Knowledge about COVID-19 spread among remote communities by respondent type, including illustrative quotes

Health center staff	Community-based volunteers	Community members
Airborne droplets: inhaled; fluids pass through the air Person-to-person Infected person coughs/sneezes Touching and handshakes: holding or shaking hands; touching contaminated surface; hugging	Sitting close: lack of spacing; crowds Infected person coughs/sneezes: saliva falls on uninfected person; when not wearing a mask Person-to-person Not wearing a mask Poor hand hygiene: not washing hands; holding or shaking hands; touching other, self, or surface Airborne: disease/fluids pass through the air	Poor hand hygiene: not washing hands; holding or shaking hands; touching other, self, or surface; hugging; greeting Sitting close: not social distancing; squeezing; gathering Infected person coughs/sneezes: through coughing, sneezing, talking, or breathing; saliva droplets; passes through the air Not wearing a mask: exchanging air
Illustrative quotes “It is transmitted through droplet infection from one person to another… It means you can acquire it through getting the fluids that are passed in the air through breathing… that is why it is advised that we sit one meter apart because it’s believed that the fluids that are coming out of the body through the airway cannot fly one meter.” – Health center staff, male, Pemba district, round 1 “This disease is transmitted through the air from one who has it through sneezing meaning those droplets will go to another person. Or touching where the person with the disease touched and handshakes and gathering in meetings or in large numbers.” – Community-based volunteer, male, Choma district, round 2 “We infect each other if we sit close to each other that we exchange the same air.” – Community member, female, Nyimba district, round 1 “The disease for COVID-19 spreads if you cough near where your friend is without covering yourself or wearing a mask the way I am wearing it now. The saliva can splash and then that person can contract that disease. Even hand shaking without protecting yourself, without applying hand sanitizer, we do not need to handshake because you can contract that disease.” – Community member, female, Pemba district, round 2		

Asymptomatic transmission was not discussed during the first round of data collection. Respondents were specifically asked about asymptomatic transmission during the second round of IDIs; they widely agreed that asymptomatic individuals can spread COVID-19. Although multiple individuals could clearly explain asymptomatic spread, some using transmission of HIV as an example (see illustrative quotes below), many could not clearly explain the topic, sometimes explaining pre-symptomatic states or being symptomatic without knowing it was COVID-19. Some community members had divergent views, stating that COVID-19 could not be spread by an individual without symptoms.

*“There are actually what we call asymptomatic patients. These are the patients who would have the virus without showing any signs and symptoms. But through the same respiratory contact, they can easily spread it to another person.” – Health center staff, male, Kalomo district, round 2*

*“[Referring to asymptomatic spread…] I will give an example. A person with HIV can live like they do not have this disease, but they will be moving around sleeping with women and they will be transmitting it. It will not stop the women from contracting it so, even a person before they reach a point of being symptomatic, they are able to spread it. That is even the women can then spread it even more because people will trust that they do not have the disease meanwhile they already have it.” – Community-based volunteer, male, Pemba district, round 2*

*“Yes he is able to spread it [COVID-19] to his friends if he frequents crowded places, even if he doesn’t have the signs.” – Community member, female, Choma district, round 2*

*“A person cannot spread it [COVID-19] if he or she does not sneeze.” – Community member, male, Choma district, round 2*

#### Prevention guidelines.

Respondents from all groups widely discussed that MoH prevention guidelines include handwashing or the use of hand sanitizer, the need to sit at least 1 m away from others (social distancing), the wearing of face masks, and limiting gathering sizes.

#### High-risk activities.

Discussion of high-risk activities revolved around attending gatherings and being in crowded locations (Table 3). Respondents specifically mentioned funerals, church services, weddings, football (soccer) matches, markets, shops, buses, and community meetings as examples of such crowded settings. Respondents also widely discussed failure to follow preventive measures as high-risk activities, including not wearing a mask, not distancing, and having poor hand hygiene. Some CBVs and community members discussed travel or movement from place to place as a high-risk activity.

**Table 3:** Knowledge of high-risk activities associated with COVID-19 among remote communities by respondent type, including illustrative quotes

Health center staff	Community-based volunteers	Community members
Gatherings and crowds: examples: funerals, football matches, church, weddings, markets, other community meetings Not following prevention guidelines: not social distancing, not wearing masks, not handwashing	Gatherings and crowds: examples: funerals, churches, football matches Not following prevention guidelines: not social distancing, not wearing masks, not handwashing Continuing to shake hands and hug Travel: movement from place to place	Gatherings and crowds: includes sitting squeezed and not spacing; examples: church, shops, weddings, funerals, buses Not following prevention guidelines: not social distancing, not wearing masks, not handwashing Continuing to shake hands and hug Travel: movement from place to place
Illustrative quotes “The high-risk activities [are] where you gather as a crowd and there is no social distancing, there is no proper hygiene like handwashing or face masking. If you do not do those things and if one in that group is infected, then it is possible that most of the people can also get infected.” – Health center staff, male, Nyimba district, round 1 “When finding yourself in overcrowded places, you are actually propagating the spread of Covid-19. Generally, I would say at funerals people are not observing social distancing, they are not masked up. And in places where people are watching soccer. Market places, those are high risk areas too.” – Health center staff, male, Choma district, round 2 “When we sit in a large group together without social distancing, then the disease can spread very fast. Also not wearing a mask on the mouth, then this disease can spread to you.” – Community-based volunteer, female, Pemba district, round 1 “By not adhering to the guidelines which are given by health worker like social distance, meeting in numbers and not wearing of masks. This is what can cause the disease to spread more.” – Community member, male, Kalomo district, round 2		

### Motivation: motivators and demotivators for performing prevention behaviors.

#### Motivators.

A number of personal and social factors emerged as motivators for good adherence to prevention methods (Table 4). Fear of contracting the disease and the fact that it can cause death featured prominently in all discussions, particularly among community members who frequently discussed hearing the growing numbers of reported illness and death in Zambia on the radio or television. Similarly, the desire to protect oneself and family was also widely discussed as a reason to follow prevention methods.

**Table 4:** Motivators for adherence to COVID-19 prevention behaviors among remote communities by qualitative theme and respondent type

Health center staff	Community-based volunteers	Community members
Personal Fear deadliness of disease: feel at risk in their area, fear of death Social Local gatherings inspected: health center staff routinely observe public places and community meetings to ensure prevention measures are followed; specifically, schools and churches Adherence enforced at clinic: to receive health services	Personal Fear deadliness of disease: there is no cure; feel at risk Social Local gatherings inspected: health center staff and volunteers ensure guidelines are being followed Adherence enforced in certain settings: in schools, at clinics, at church, in meetings	Personal Fear deadliness of disease: fear contracting the disease; feel at risk; hear about growing case numbers in Zambia from the radio/tv Protect themselves: prevent themselves from contracting the disease; do not want to get sick; maintain their health Social Closed doors: began adhering to guidelines when schools, churches, and businesses began closing; realized seriousness of disease Adherence enforced in certain settings: in schools, at clinics, at church
Illustrative quotes “The environmental health technician usually goes to inspect the churches to make sure that people have put the required equipment in place, like hand washing facilities, that people are masking up, that people are also spacing up.” – Health center staff, female, Kalomo district, round 1 “[Community members] have come to believe now that COVID-19 is real and they have come to understand it.” – Health center staff, female, Pemba district, round 2 “Here at the health facility, when you come without a mask, they would send you to go back and buy a mask. [Only then] they will come and give you the drugs, that is when you’ll be attended to.” – Community-based volunteer, female, Nyimba district, round 1 “At the under-five clinic, we see that there is water, in schools we see that there is water and children wash their hands and are wearing masks. Even at church they worship from outside, they do not worship from inside.” – Community-based volunteer, female, Kalomo district, round 2 e. “We follow these guidelines to protect our own lives. When we follow those [guidelines], we cannot get sick. So we protect ourselves from the coronavirus.” – Community member, female, Nyimba district, round 1 “They follow because even in schools’ pupils stopped learning the way they used to learn, they just learn for an hour, and they knock off while wearing a mask.” – Community member, female, Choma district, round 2 “We have heard about death, thousands and thousands of people have died and in the past days when news is being read, they mention the number of people who have died because we saw that churches were closed, okay most of the things were closed. We just used to say at home and worship from there.” – Community member, female, Choma district, round 2		

Community members described the closing of schools, churches, and businesses as influencing community adherence to the COVID-19 prevention measures. Additionally, health center staff and CBVs described the inspection and monitoring of local gatherings, particularly early in the pandemic, to ensure adherence to prevention methods in high-risk settings. These inspections are frequently conducted by EHTs and occurred at church services, schools, or other public spaces and events. The EHTs checked the availability of handwashing stations with soap and water, that attendees were wearing masks and social distancing, and that the number of attendees adhered to restrictions.

All respondent groups described higher adherence rates in some settings where guideline adherence was “required” or more strictly enforced. Schools, clinics, and churches were repeatedly discussed among all respondent groups. School children were required to wear face masks, and classes were split in size to reduce indoor crowding. At clinics, patients were required to wash their hands, wear masks, and socially distance to receive services. Health center staff described turning away individuals not adhering to these behaviors or sending them to a local shop to procure a face mask when necessary. Church leaders also enforced guideline adherence more than other settings, such as bars and businesses, and ensured handwashing stations were available.

#### Demotivators.

All major demotivators to performing prevention behaviors discussed by respondents were at the personal, rather than the social, level (Table 5). Generally, respondents from all groups discussed people’s doubt of the disease’s existence as a major reason for non-adherence to prevention behaviors during both data collection timepoints. The perceived lack of local cases and deaths first in Zambia then within their very remote, rural communities perpetuated doubt among some community members, making some believe COVID-19 was not a threat. Respondents also discussed individuals believing the disease to be a myth, underestimating its severity, being determinedly ignorant, or believing that the information about COVID-19 was fabricated as a political tool for the upcoming election (in mid-2021). Other respondents described community members as experiencing pandemic fatigue (i.e., discomfort and resistance to mask wearing; tired of adhering to guidelines or hearing about COVID-19; asserting that COVID-19 is not worth the concern, believe COVID-19 has ended and is no longer a risk, etc.). Respondents frequently expressed pandemic fatigue among community members concurrent with their statements over doubt of disease severity, geographic irrelevance, and disbelief in need for adherence measures.

**Table 5:** Demotivators for adherence to COVID-19 prevention behaviors among remote communities by qualitative theme and respondent type

Health center staff	Community-based volunteers	Community members
Personal Doubt/fatigue about existence of COVID-19: have not seen a case firsthand; have become fatigued of hearing about COVID-19 and prevention measures; do not believe COVID-19 is a threat Lack knowledge: do not understand certain aspects including purpose of prevention measures, infectiousness, lethality, the need to perform prevention behaviors in all settings	Personal Doubt/fatigue about existence of COVID-19: have not seen a case firsthand; do not believe COVID-19 is a threat; think the risk is over because COVID-19 has ended Lack of knowledge: do not understand why they need to continue adhering to guidelines	Personal Doubt existence of COVID-19/benefit of prevention measures: think COVID-19 is a lie; do not believe it is in Zambia; have never seen it firsthand; feel detached from it because it is a faraway problem; underestimate its severity; doubt due to lack of knowledge, ignorance, or stubbornness; believe COVID-19 is linked to politics and upcoming election Alcohol: fail to adhere to guidelines when drinking
Illustrative quotes “I think [community members] don’t understand social distancing because [health center staff] have kept on talking about social distancing but I have observed that whenever there is a wedding, whenever there is a funeral, normally people are actually grouped together without social distancing. So, I think we still have a problem where social distancing is concerned.” – Health center staff, male, Kalomo district, round 1 “For those that believe they can’t get COVID-19, it’s because they’ve made up their minds that COVID-19 is not there. [They think] the government and the world have just made it up. It doesn’t exist. They don’t believe that they can have something that doesn’t exist. Then with those that believe that they can get it, we’ve been highlighting on how the disease has been trending. For the others, we show them videos of actual patients. There are some people who only believe when they see something [in person], so when they see those videos, they strongly believe and try to use all the interventions to protect themselves.” – Health center staff, male, Nyimba district, round 2 “The biggest barrier for people not to adhere to the guidelines is not believing or not accepting that this pandemic is real.” – Community-based volunteer, female, Nyimba district, round 2 “People are just stubborn. Because they have never seen [a case of COVID-19 or] what the disease does.” – Community member, female, Choma district, round 1 e. “It is drunkards who are found in taverns. They are the ones who do not follow the guidelines because they meet anyway, and they sit [together]. If they are normal and not yet drunk, they will be wearing a mask, but when they reach the tavern and start drinking, they put the mask in their pocket.” – Community member, female, Kalomo district, round 2		

Health center staff and CBVs noted early challenges with adherence when COVID-19 was first detected in Zambia when understanding was low and many individuals lacked knowledge. Lack of knowledge was a stronger theme during round 1 but persisted during round 2. During round 1, health center staff were particularly concerned that many in the community still did not understand some of the most critical aspects of COVID-19, including how the guidelines linked to modes of transmission meant to prevent spread of the virus, how infectious the virus was, and how lethal it could be if widespread transmission occurred.

Many community members and a few health center staff and CBVs discussed the role alcohol played in failure to adhere to prevention behaviors. Individuals at drinking establishments were described as sharing glasses, not wearing masks, not social distancing, and not washing hands. They were described as forgetting about prevention behaviors as soon as they began consuming alcohol. A few respondents also described high emotional states, such as excitement or grief (i.e., weddings and funerals), affecting adherence to prevention guidelines.

### Behavioral skills: skills to perform prevention behaviors correctly and at the right times.

#### Hand hygiene.

Although nearly all respondents cited hand washing as an important aspect of COVID-19 prevention, few discussed proper handwashing technique (Table 6). Additionally, the use of soap was frequently—though not universally—discussed, particularly by health center staff and CBVs. Only a few community members specifically mentioned soap when discussing handwashing. Respondents frequently discussed the timing of handwashing, including washing hands upon arriving to and leaving a gathering and after touching a foreign object (e.g., money, surfaces) or another person. Having handwashing stations at gatherings or in public spaces was discussed as a prerequisite for gatherings.

**Table 6:** Behavioral skills (technique and timing) related to COVID-19 prevention guidelines among remote communities by qualitative theme and respondent type

Themes	Health center staff	Community-based volunteers	Community members
Hand hygiene	Technique: must use water and soap or hand sanitizer Timing: arriving to or leaving gathering; after touching a foreign object or another person	Technique: must use water and soap or hand sanitizer Timing: arriving to or leaving gathering; after touching a foreign object or another person	Technique: must use water and soap or hand sanitizer Timing: arriving to or leaving gathering; after touching a foreign object or another person
Face masks	Technique: wear over mouth / nose and mouth Timing: wear in crowded locations	Technique: wear to trap infected droplets and keep them in/out Timing: wear in crowded locations	Technique: Cover mouth with mask Timing: wear in crowded locations
Social distancing	Technique: need to sit at least one meter apart from anyone; germs can’t reach another person one meter away Timing: sit distanced especially when in crowds	Technique: need to sit at least one meter apart from anyone; saliva can’t reach another person one meter away; don’t sit squeezed Timing: sit distanced especially when in crowds	Technique: maintain a one-meter distance from others Timing: important not to sit squeezed during gatherings
Limiting gatherings	Technique: limit number of attendees; ventilation important; must maintain distancing & adhere to guidelines	Technique: limit number of attendees; maintain distancing & adhere to guidelines	Technique: limit number of attendees; must maintain distancing & adhere to guidelines
Illustrative quotes “You need to have a meter from where your friend is. That is the specification which was given in terms of social distancing.” – Health center staff, male, Nyimba district, round 1 “[The guidelines are to] mask up, avoid overcrowded places, proper hand washing, avoid unnecessary movements, and avoid traveling when you’re not feeling too well.” – Health center staff, female, Pemba district, round 2 “The face masks are supposed to be worn because the virus uses that route. There should not be exchange of air. If the person has the coronavirus and is talking to the person who does not have, the person who is not wearing a mask can easily contract it.” – Community-based volunteer, female, Kalomo district, round 1 “Social distance is also necessary when you have meetings – sitting well-spaced. Also washing hands. We are supposed to be washing hands. They teach the way we are supposed to be washing our hands so that we prevent this disease. Then we were advised that every time we have meetings, we should be wearing masks, so that we can prevent this disease. The health facility has a big role in guiding us, because every time we are in meetings, they talk about all this.” – Community-based volunteer, male, Kalomo district, round 2 e. “Other guidelines from the government are that we are not supposed to gather not more than 50 people in one place. Again, we are not supposed to gather with a lot of people for more than 2 hours.” – Community member, female, Pemba district, round 1 “[The guidelines are] washing hands when you enter a shop. When you gather in numbers, you need to wear a face mask so that you don’t catch the disease. And sitting 1 meter apart.” – Community member, female, Kalomo district, round 2 “When I reach other people’s homes, they have put a bucket of water at the road, for washing the hands with soap. Even in a shop when I want to enter, water is also there. Then also I am supposed to wear a mask on the face.” – Community member, female, Kalomo district, round 2 “The district emphasizes that we should avoid social gatherings. We should sanitize our hands and wash them. Then we should wear masks whenever we are moving, going somewhere, or whenever we want to attend any [gathering]... So those are the regulations that are coming from the district.” – Community member, female, Nyimba district, round 2			

#### Face masks.

Some respondents noted the involvement of both the nose and mouth in spreading COVID-19. For example, community members frequently described the risk of an infected person touching their nose and then shaking hands with someone. However, very few respondents discussed the proper technique of wearing a face mask as covering both the nose and mouth. Respondents generally talked about face masks “covering the mouth” or more vaguely mentioning wearing a face mask as a necessary precaution.

All respondent groups discussed wearing face masks as particularly important when in gatherings or crowded areas or when social distancing was not possible. Respondents frequently made the link with the modes of transmission, explaining that face masks keep respiratory droplets (or saliva) in (for an infected individual) or out (for an uninfected individual).

#### Social distancing.

Respondents widely discussed the need to sit at least 1 m away from others (some implying a person outside one’s household) to limit the spread of COVID-19 because the droplets or “saliva cannot travel more than one meter.” The need for social distancing was especially emphasized when in crowded locations. Emphasis was often on social distancing when sitting. CBVs and community members often described this as the importance of “not sitting squeezed.”

#### Avoiding gatherings/high-risk settings.

Respondents consistently discussed the need to follow all mask wearing, social distancing and hand hygiene guidelines while in high-risk settings, which were universally linked with gatherings and crowded places. However, there was almost no discussion of communities choosing to avoid high-risk settings, such as not going to church, football matches, or funerals due to COVID-19 aside from when these gatherings were specifically closed by the government in the interest of public safety. Some respondents mentioned the need for gatherings to be short, lasting only 1 or 2 hours.

When asked about gathering indoors, the majority of health center staff emphasized the role of proper ventilation to keep air circulating and limit disease spread. Although only a few CBVs and community members noted the detriment of all participants “breathing the same air,” many emphasized that the meeting space must be large enough to maintain distancing provisions. Specific locations, like homes and clinic rooms, were discussed as having little space and poor ventilation, unlike outdoor events. For schools and churches participants discussed them having sufficient space and ventilation but would require restricted group size.

### Behaviors: perceived performance of COVID-19 prevention behaviors.

Although many respondents believed communities were generally adhering to COVID-19 prevention guidelines, there was widespread acknowledgment that this was not always the case. Respondents noted that adherence was highest at specific kinds of institutional settings, including clinics, health outreach sites, churches, and schools (Table 7). During discussions, respondents linked the practice of prevention behaviors in these settings to enforcement efforts by health center staff and other community leaders and that these gatherings usually begin with short IEC on COVID-19, which serves as a reminder to all present participants. These locations (excluding clinics) were closed during times of peak infection rates in Zambia. Mask wearing, social distancing, and handwashing were more strictly enforced; handwashing stations were consistently provided.

**Table 7:** Perceived levels of adherence in specific locations discussed by qualitative respondents

Locations of greater perceived adherence	Locations of lower perceived adherence
Clinics Health outreach sites Churches Schools	Funerals Football matches Weddings Taverns/bars Markets Within own homes

Respondents widely discussed non-adherence as highest in specific types of informal settings outside of formal, institutional settings (as mentioned above). Funerals, football matches, weddings, and bars were discussed as high-risk settings where the spread of the virus was more likely, as people gathered with fewer participants practicing mask wearing or social distancing. Attendance at these events was generally discussed as being very high, particularly for funerals, which could draw 300–500 mourners. Handwashing stations were also described as being less consistently provided in these settings, and alcohol was often consumed. Markets and individual’s homes were additional locations described as having low adherence. Illustrative quotes are included below.

*“Here at the health facility, when you come without a mask, they would send you to go back and buy a mask. [Only then] they will come and give you the drugs, that is when you’ll be attended to.” – Community-based volunteer, female, Nyimba district, round 1*

*“At funerals, people don’t pay attention to the guidelines. They were squeezed [close together]. They were not wearing the face masks. The reason why it was hard for people to follow the guidelines at this moment is because they are grieving. They tend to forget everything else because of that thing that has happened.” – Community-based volunteer, female, Pemba district, round 1*

*“The place where I see that they follow [the guidelines] is at the churches. Because there the leaders emphasize a lot that we should be following the guideline. They say that everyone should wash their hands. We don’t sit the way we used to sit in the past close to each other, there is spacing [now].” – Community member, female, Nyimba district, round 2*

*“I saw that people did not follow these guidelines the times when we have weddings. Because [they are] happy…they do not follow the guidelines. There is no mask [wearing], no washing hands, just mingling. Then also the time of ball games. The footballers just come…they are singing…they mingle, they don’t follow the guidelines. Also like in the beer halls…[people] don’t even follow the guidelines.” – Community-based volunteer, male, Choma district, round 2*

*“The places where they don’t follow the guidelines sorry in sorrow situations like in funerals. In funerals, when you count the people, maybe they are about five hundred, maybe those who have masks are just fifty if they are a lot. All these they do not have masks. So in funerals they don’t follow the guidelines strictly.” – Community member, female, Choma district, round 2*

### Moderator: access to resources needed to perform prevention behaviors.

Many respondents discussed community members not having sufficient resources to protect themselves, such as being unable to afford masks. Some respondents described classes to teach community members how to sew their own masks from pieces of cloth.

Hand sanitizer was widely described as being out of reach for many community members. Many respondents mentioned communities substituting wood ash for handwashing when soap was not available, implying that accessing soap can be a frequent challenge in these rural communities. Although the need to have water buckets and soap at gatherings and in public locations was widely acknowledged by all respondent groups, many reported that handwashing stations were not always available. Respondents stated that guideline adherence, specifically to handwashing, was highest in settings where handwashing basins or buckets were readily available for all to use, including at clinics, churches, and businesses; these resources were often not found during gatherings in less institutional settings, such as football matches and funerals. Illustrative quotes are included below:

*“What we may need as a facility are the protective equipment which are supposed to be worn when there is a case found. Now money is a challenge so sometimes we run out of hand washing soap. Sometimes people wash their hands with just water. Sometimes we run out of hand sanitizers. Sometimes the district sends to us… Then the other thing is we were using chitenge material [as masks] for protection which I feel was not fully protective. But at the moment we have surgical masks.” – Health center staff, female, Choma district, round 1*

*“[Discussing prevention guidelines…] There is washing of hands with soap. If you do not wash your hands with soap you use hand sanitizer. Some do not have hand sanitizer and soap meaning they have to use water and ash.” – Community-based volunteer, female, Kalomo district, round 2*

All groups agreed that community members were more likely to adhere to guidelines when resources such as handwashing basins, face masks, and soap were made available to them.

### Moderator: information, education, and communication dissemination efforts.

Respondents generally attributed strong community understanding about COVID-19 to widespread sensitization efforts that began soon after the pandemic was declared (Table 8). Two main sensitization methods were described: 1) health education at gatherings or meetings and 2) messaging over the radio. According to the majority of respondents, health education was provided to community members at nearly any gathering they attended. This included during clinic visits and normally scheduled health education sessions at the health centers or in communities, at schools, during weekly church services, and at other ad hoc village meetings. Health education was provided by health center staff, CBVs, or other community leaders, including individuals in religious, civic, and traditional leadership positions who had been oriented by the health center staff. Respondents explained that the information provided during these health education sessions included basic information about the disease as well as prevention measures and government guidelines.

**Table 8:** Methods and content of COVID-19 information, education, and communication dissemination to remote communities by qualitative theme and respondent type

Themes	Health center staff	Community-based volunteers	Community members
Methods	Teach whenever community gathers: at the health center, health outreach, schools, and churches Educate other community leaders: involve CBVs, religious, civic, and traditional leaders to disseminate information in their villages	Teach whenever community gathers: at church, schools, and other community meetings Lead by example: along with explaining guidelines, encourage others to follow by showing yourself following guidelines	Education sessions during community gatherings: at the health center, health posts, churches, schools, community meetings. Radio: announcements and government messaging
Content	Provide basic information: education on disease signs, symptoms, and prevention methods; distribute IEC materials	Teach about prevention and self-protection: Explain spread, prevention methods, and high-risk activities; warnings about lack of cure and deadliness of disease	Taught basic information: existence of COVID-19 and prevention methods; deaths in Zambia and internationally; government messaging
Illustrative quotes “We [health center staff] are using the community-based volunteers too…We equip them and then we send them out to go and teach on our behalf. We [health center staff] have also gone in all the schools to teach the school staff, to teach the pupils on the preventive measures and what COVID-19 is all about. We also involved the pastors, the church leaders, and the civil and the civic leaders that is the counsellors and the headmen and the alike. We had to bring them together teach them on COVID-19 so that they could also help us disseminate the information in the communities. ” – Health center staff, male, Kalomo district, round 1 “Wherever there are gatherings the message about COVID-19, I do not leave it out when talking. When we gather… even in churches, I am one of those sensitizing about COVID-19. Wearing masks, washing hands, social distancing, not holding hands, not greeting. So we spread this message well.” – Community-based volunteer, female, Pemba district, round 1 “Information about COVID-19 is spread whenever you gather or if there is a meeting. Wherever we gather, they announce that this disease kills.” – Community member, female, Kalomo district, round 1 “We first heard in the radios that there is COVID-19. Then from the radio, then again the health personnel came and also told us. Then, when the health personnel finished telling us, they started moving in the churches, and in schools, saying if the children should not be using masks, then the schools will be closed. So, that’s when we started seeing that COVID-19 is really there. That’s how we heard the message.” – Community member, male, Pemba district, round 2 “I heard that in Lusaka and in Monze that people have died of Covid-19. I heard on the radio they were announcing.” – Community member, female, Pemba district, round 2			

Many community members also discussed hearing about COVID-19 through announcements on the radio. They reported learning about the existence of COVID-19, hearing news reports about deaths from COVID-19 in Zambia and internationally, and hearing government messaging about prevention.

## DISCUSSION

A scoping review of knowledge, attitudes, and practice (KAP) studies found that high knowledge of COVID-19 was not always associated with positive attitudes or adherence to prevention behaviors^30^; we posit that additional factors, such as motivations, behavioral skills, and moderators, may influence individual’s practice of prevention behaviors. Through the frame of the IMB model, we described the levels of information rural Zambian communities have about COVID-19, their motivations for adhering to prevention methods, and the skills required to perform the prevention behaviors. We conducted FGDs with remote, rural communities in Southern and Eastern Provinces Zambia that are generally poor; have limited education; and have limited access to internet, social media, and television.^31^ We also interviewed health systems staff and CBVs who routinely interact with these remote communities, provide them with health services and information, observe their behaviors, and oversee enforcement of the COVID-19 prevention guidelines.

### Information and behavioral skills.

Generally, all respondent groups showed good knowledge of COVID-19, consistent with KAP studies in sub-Saharan Africa.^30^ It appears that IEC dissemination efforts were successful in reaching the most rural Zambian individuals in this study. However, some gaps in respondent knowledge and skills existed, such as no discussion of loss of taste/smell as a potential COVID-19 symptom and unique presentation of this disease.^32^ There was also little mention of wearing masks over the nose and mouth as preventive measures. Similar knowledge gaps have been found in other sub-Saharan African populations, highlighting the need to continue to adapt IEC materials to identified gaps and the need to design specific messaging to target particularly vulnerable or particularly non-adherent populations.^33^

### Personal experiences and sources of information.

Personal experience with COVID-19 was a particularly important motivator for performing prevention behaviors, with an individuals’ fear versus doubt of the disease raised as a critical deciding factor in adherence. Misinformation spreading doubt about the severity of COVID-19 or need for prevention behavior adherence has been reported in many African communities.^34^

Respondents in this study discussed non-adherence as particularly widespread when case numbers were still low in Zambia, with adherence to prevention behaviors increasing as case numbers grew and media coverage of daily cases and high-profile deaths expanded. Due to the rural, impoverished nature of these areas, these communities have limited access to internet and television, lacking access to widespread national information campaigns on these platforms.^6^^,^^14^ Consequently, these remote communities likely received fewer updates on the state of the pandemic nationally or globally from fewer kinds of sources. One study in Hong Kong during the first months of the COVID-19 pandemic suggested that receiving information from a greater number of sources can result in more concern over COVID-19 and better adherence to prevention behaviors.^35^ Other studies have linked lack of phone or internet access with limited COVID-19 knowledge; however, COVID-19 knowledge, attitudes, and practice surveys have been primary conducted over the phone in sub-Saharan Africa, limiting the ability to assess this link in greater depth.^30^ Furthermore, official figures of cases and deaths in Zambia, which were disseminated widely by government and news sources, may have underestimated the true impact of the national epidemic, particularly early in the pandemic when many deaths likely occurred outside of the formal health system, as recent studies have suggested.^36^^,^^37^

Even with these challenges and limitations, the Government of Zambia was able to reach and educate many remote-living individuals by implementing television, radio, and social media campaigns and ensuring that health center staff, CBVs, and community leaders provided widespread sensitization. This is common practice for sharing health education in LMICs and could be more broadly applicable for reaching geographically remote or otherwise vulnerable and hard-to-reach populations globally.

### Resources.

Although knowledge was generally widespread within these rural communities, lack of resources remained a persistent barrier, a common theme in many areas of life particularly in rural areas of LMICs. Although community members knew the importance of hand hygiene, they were not always able to perform the behavior correctly. Soap, universally recognized as a critical piece for performing hand hygiene behaviors, was noted by some respondents as a challenge, with ash being used as a substitute. Although ash has been recommended by the WHO as a substitute for soap in emergencies,^38^ there is scientific uncertainty on its impact on disease rates^39^ and no evidence on its effectiveness against the SARS-CoV-2 virus. Although providing household-level substitutions is likely acceptable, providing soap resources should be prioritized for locations with the highest risks of transmission, including clinics, churches, and other places of gathering. Masks were similarly described as financially unattainable by many. Mask-making classes were described by some respondents and could make masks more attainable to vulnerable households while reserving medical-grade personal protective equipment for health workers and other high-risk individuals. Even with perfect information, motivation, and skills, behaviors cannot be performed if individuals do not have the resources to do so.

### Institutional versus non-institutional settings.

Although respondents were largely positive when discussing community-level adherence to COVID-19 prevention methods, they repeatedly described instances of widespread non-adherence within these rural communities. Social motivators appeared to play a major role in general community adherence, particularly in high-risk settings. Institutional settings, including clinics, church services, and meetings with traditional leadership, were described as experiencing higher levels of perceived adherence among the community at large. Institutional settings generally had stronger adherence enforcement measures in place and had clear leaders who supported guideline adherence. Additionally, respondents explained that visits to institutional settings frequently began with COVID-19 sensitization sessions, which systematically reinforces messages community members may have heard through other channels.

Informal, non-institutional community settings, such as at weddings, funerals, and football (soccer) matches, often lacked similar social expectations and leaders to model or enforce adherence. These settings were described as problematic and often had lower levels of general adherence for hand washing, mask wearing, social distancing, and restricted group sizes. Although health center staff and CBVs discussed repercussions for individuals not following prevention guidelines at the clinics (refusal of services), churches (revoking permits for gatherings), or meetings with traditional leaders (disbanding and rescheduling), such repercussions were not discussed for these other informal community events. These findings are consistent with concurrent quantitative data from these same communities, which found that rural Zambian participants reported attending large gatherings throughout the pandemic; higher levels of prevention behavior adherence were perceived at clinics and churches, whereas funerals experienced larger crowds with much lower levels of reported mask wearing among participants.^28^ The average funeral attendance was 200–300 people, and attendees were 55% less likely to report wearing masks than those attending a clinic visit.^28^

In addition to low social pressure for adherence, the high (positive or negative) emotions of the crowds at informal community events may override personal-level motivators for adherence and perceived risk. A few respondents discussed non-adherence in this way, stating that joy at weddings and football matches and grief at funerals made attendees “forget” about COVID-19 and the need to practice prevention behaviors in these settings. Similarly, long-engrained cultural expectations around physical touch while greeting (i.e., shaking hands, hugging), particularly in emotional or ceremonial settings, could potentially override perceived risk. A systematic review of COVID-19 risk communication and community engagement in 13 African countries acknowledged widespread resistance to attendance guidelines at funerals and resistance to stop shaking hands or hugging in many communities.^40^ With widespread acknowledgment of these locations of low adherence, corroborated by recent quantitative findings^41^, funerals have the potential to become superspreading events in Zambia, particularly with the increasing transmissibility of COVID-19 variants. Other sub-Saharan African countries with similar cultural traditions (i.e., large village funerals of multiple hundreds of attendees) have highlighted the likely role of funerals in the community spread of COVID-19.^42^^–^^46^

During a resurgence of COVID-19 or during a potential future pandemic, efforts to increase community-level adherence to prevention guidelines should target these informal, non-institutional, low-adherence community events that continued to occur outside of regulations. Although respondents repeatedly stated that more information dissemination was needed to convince doubters and outstanding non-adherers at these informal, non-institutional community events, this is unlikely to have drastically changed behaviors because lack of information did not seem to be a major influencer of non-adherence here or in other KAP studies.^30^ Yet, health personnel repeatedly attributed community non-adherence to lack of knowledge. If lack of knowledge is not the major driver of non-adherence as they believe, but rather the social and contextual motivators were a more critical motivators of widespread adherence, as community members themselves highlighted, opportunities to affect non-adherence in settings where it was still widely occurring may have been missed.

On the other hand, increasing social pressure through greater enforcement measures and provision of needed supplies (i.e., handwashing stations) could have more widely affected general adherence in these informal settings and affected potential transmission occurring during these community gatherings. Engaging with local leaders in religious, civic, and traditional leadership positions has been shown as critical for many health-related interventions^47^ and was practiced with success in multiple African countries to address COVID-19.^40^ Assigning community leaders to monitor high-risk, low-adherence settings could influence community behaviors without adding additional monitoring duties to EHTs and an already overburdened, resource-limited health system.

### Strengths and limitations.

Although this was a purely qualitative study, it included a rigorous design with a large sample size of respondents at different levels who confirmed each other’s responses at two different points in time. Furthermore, the qualitative results were interpreted against the IMB model, a well-known socio-behavior model from the HIV literature that has only been posited once before for use with COVID-19, making this analysis a unique contribution of the literature.^48^ However, this study has several limitations. First, as with much of the world, the COVID-19 situation in Zambia has continued to evolve. Data collection occurred just prior to the more widespread and deadly third wave in Zambia, so the findings may not reflect well the current behaviors of rural communities, which likely experienced more direct effects from these surges, potentially affecting their motivations and influencing their behaviors.

Second, responses from participants may have been subject to social desirability bias. Adherence to guidelines may have been emphasized, whereas unfavorable views and non-adherence may have been downplayed. We attempted to limit the likelihood of social desirability bias in the data by asking questions “about the community” instead of about the respondents themselves. Because the data sources are IDIs and FGDs, we are only able to report on what people perceive. Additionally, respondents were not asked to demonstrate proper prevention behavior techniques. Observational data would be needed to confirm accurate performance of these behaviors, determine actual adherence rates in various settings, and confirm reported perceptions.

Additionally, approximately one-third of the participating community women received additional health education around COVID-19 twice monthly through the ECD intervention. However, we did not find any noteworthy differences between the overarching study arms. Nonetheless, caution should be taken when generalizing these data outside of the immediate communities from which they were collected.

Lastly, the qualitative instruments did not include questions on COVID-19 vaccine perceptions or uptake due to the timing of data collection. During our first round of data collection (November 2020), COVID-19 vaccines were still in testing internationally and had not yet been distributed. During the second round of data collection (April/May 2021), the first shipments of vaccines were only just reaching Zambia and were concentrated in urban areas. At the time of instrument development, ethics approval, and data collection, there was no vaccine distribution within our study areas. Futher information on COVID-19 vaccine perceptions and uptake among these rural community members and stakeholders is important.

## CONCLUSION

Assessing the factors associated with COVID-19 prevention behaviors adherence in a rural Zambian population has revealed generally strong understanding of COVID-19 symptoms, spread, and high-risk activities with some knowledge and skills gaps that suggest the need for additional targeted messaging. The findings suggest that performance of prevention behaviors is driven by personal and social factors, that the different factors can motivate or demotivate adherence, and that behaviors are also affected by epidemic severity and its associated media coverage. Additionally, our findings highlight specific high-risk settings where perceived community-wide adherence is lowest: informal, non-institutional settings without clear community leaders, including funerals, weddings, and football games. Based on the responses about motivators and demotivators to adherence, specific interventions can be designed to target those settings where the potential for rapid spread of COVID-19 is greatest.

## References

[1] WHO , 2020. WHO Director-General’s Opening Remarks at the Media Briefing on COVID-19 - 11 March 2020. Geneva, Switzerland: World Health Organization. Available at: https://www.who.int/director-general/speeches/detail/who-director-general-s-opening-remarks-at-the-media-briefing-on-covid-19–-11-march-2020. Accessed March 28, 2021.

[2] World Development Indicators Database , 2021. CountryProfile | World Development Indicators. Washington, DC: World Bank. Available at: https://databank.worldbank.org/views/reports/reportwidget.aspx?Report_Name=CountryProfile&Id=b450fd57&tbar=y&dd=y&inf=n&zm=n&country=ZMB. Accessed March 28, 2021.

[3] WHO , 2022. WHO Coronavirus (COVID-19) Dashboard with Vaccination Data. Geneva, Switzerland: World Health Organization. Available at: https://covid19.who.int/. Accessed July 29, 2022.

[4] Chipimo PJ , 2020. First 100 persons with COVID-19-Zambia, March 18-April 28, 2020. Morb Mortal Wkly Rep 69: 1547–1548. 10.15585/mmwr.mm6942a5 PMC7583505 33090982

[5] Ministry of Health, Zambia National Public Health Institute , 2020. *Zambia COVID-19 Situational Report No. 01*. http://znphi.co.zm/publications-2/. Accessed April 12, 2021.

[6] Ministry of Health Zambia , 2022. *Ministry of Health Zambia | Facebook*. https://www.facebook.com/mohzambia/. Accessed July 28, 2022.

[7] Mwenda M , 2021. Morbidity and mortality weekly report detection of B.1.351 SARS-CoV-2 variant strain-Zambia, December 2020. Morb Mortal Wkly Rep 70: 280–282. 10.15585/mmwr.mm7008e2 PMC8344984 33630820

[8] WHO , 2022. Zambia: WHO Coronavirus Disease (COVID-19) Dashboard with Vaccination Data. Geneva, Switzerland: World Health Organization. Available at: https://covid19.who.int/region/afro/country/zm. Accessed July 29, 2022.

[9] Zambia Nation Public Health Institute , 2021. Zambia (COVID-19) General Dashboard. Lusaka, Zambia: Zambia Nation Public Health Institute. Available at: https://rtc-planning.maps.arcgis.com/apps/opsdashboard/index.html#/3b3a01c1d8444932ba075fb44b119b63. Accessed March 28, 2021.

[10] WHO , 2020. Transmission of SARS-CoV-2: Implications for Infection Prevention Precautions: Scientific Brief, 09 July 2020. Geneva, Switzerland: World Health Organization. Available at: https://apps.who.int/iris/handle/10665/333114. Accessed March 20, 2021.

[11] Chu DK , 2020. Physical distancing, face masks, and eye protection to prevent person-to-person transmission of SARS-CoV-2 and COVID-19: a systematic review and meta-analysis. Lancet 395: 1973–1987. 32497510 10.1016/S0140-6736(20)31142-9 PMC7263814

[12] Clase CM Fu EL Joseph M Beale RC Dolovich MB Jardine M Mann JF Pecoits-Filho R Winkelmayer WC Carrero JJ, 2020. Cloth masks may prevent transmission of COVID-19: an evidence-based, risk-based approach. Ann Intern Med 173: 489–491. 32441991 10.7326/M20-2567 PMC7277485

[13] Czypionka T Greenhalgh T Bassler D Bryant MB, 2021. Masks and face coverings for the lay public a narrative update. Ann Intern Med 174: 511–520. 33370173 10.7326/M20-6625 PMC7774036

[14] Zambia National Public Health Institute , 2022. *Zambia National Public Health Institute | Facebook*. Available at: https://www.facebook.com/ZMPublicHealth. Accessed July 28, 2022.

[15] Li Y Campbell H Kulkarni D Harpur A Nundy M Wang X Nair H, 2021. The temporal association of introducing and lifting non-pharmaceutical interventions with the time-varying reproduction number (R) of SARS-CoV-2: a modelling study across 131 countries. Lancet Infect Dis 21: 193–202. 33729915 10.1016/S1473-3099(20)30785-4 PMC7581351

[16] Zhao S Wang K Chong MK Musa SS He M Han L He D Wang MH, 2022. The non-pharmaceutical interventions may affect the advantage in transmission of mutated variants during epidemics: a conceptual model for COVID-19. J Theor Biol 542: 111105. 35331730 10.1016/j.jtbi.2022.111105 PMC8934756

[17] Iyaniwura SA Rabiu M David JF Kong JD, 2021. Assessing the impact of adherence to non-pharmaceutical interventions and indirect transmission on the dynamics of COVID-19: a mathematical modelling study. Math Biosci Eng 18: 8905–8932. 34814328 10.3934/mbe.2021439

[18] Alvi MM Sivasankaran S Singh M, 2020. Pharmacological and non-pharmacological efforts at prevention, mitigation, and treatment for COVID-19. J Drug Target 28: 742–754. 32643436 10.1080/1061186X.2020.1793990

[19] Kwasnicka D Dombrowski SU White M Sniehotta F, 2016. Theoretical explanations for maintenance of behaviour change: a systematic review of behaviour theories. Health Psychol Rev 10: 277–296. 26854092 10.1080/17437199.2016.1151372 PMC4975085

[20] Davis R Campbell R Hildon Z Hobbs L Michie S, 2015. Theories of behaviour and behaviour change across the social and behavioural sciences: a scoping review. Health Psychol Rev 9: 323–344. 25104107 10.1080/17437199.2014.941722 PMC4566873

[21] DiClemente RJ Salazar LF Crosby RA, 2011. Health Behavior Theory for Public Health: Principles, Foundations, and Applications, 1st edition. Burlington, MA: Jones & Bartlett Learning.

[22] Fisher WA Fisher JD Harman J, 2003. The Information-Motivation-Behavioral Skills Model: a general social psychological approach to understanding and promoting health behavior. Suls J and Wallston KA, eds. Social Psychological Foundations of Health and Illness. Hoboken, NJ: Blackwell Publishing Ltd, 82–106.

[23] National Institutes of Health U.S. National Library of Medicine , 2021. Evaluation of Scaling Up Early Childhood Development in Zambia. Bethesda, MD: NIH. Available at: https://clinicaltrials.gov/ct2/show/NCT03991182?term=NCT03991182&draw=2&rank=1. Accessed March 29, 2021.

[24] Central Statistical Office Zambia , 2016. 2015 Living Conditions Monitoring Survey Report. Lusaka, Zambia: Zambia Statistics Agency. Available at: http://www.zamstats.gov.zm/report/Lcms/2006-2010LCMSReportFinalOutput.pdf. Accessed June 5, 2018.

[25] Zambia Statistics Agency , 2020. Zambia Demographic and Health Survey 2018. Lusaka, Zambia: Zambia Statistics Agency. Available at: www.DHSprogram.com. Accessed May 27, 2020.

[26] Government of the Republic of Zambia , 2014. Community Based Volunteers Skills Audit Survey Report. Lusaka, Zambia: University of Zambia. Available at: http://dspace.unza.zm/handle/123456789/5131. Accessed July 28, 2022.

[27] Windows , 2023. *NVivo 11 for Windows Help - Run a Coding Comparison Query*. Available at: https://help-nv11.qsrinternational.com/desktop/procedures/run_a_coding_comparison_query.htm. Accessed February 12, 2023.

[28] Juntunen A Kaiser JL Ngoma T Hamer DH Fink G Rockers PC Biemba G Scott NA, 2022. Lessons from a year of COVID-19 in Zambia: reported attendance and mask wearing at large gatherings in rural communities. Am J Trop Med Hyg 108: 384–393. 36509059 10.4269/ajtmh.22-0460 PMC9896318

[29] O’Brien BC Harris IB Beckman TJ Reed DA Cook DA, 2014. Standards for reporting qualitative research: a synthesis of recommendations. Acad Med 89: 1245–1251. 24979285 10.1097/ACM.0000000000000388

[30] Nwagbara UI Osual EC Chireshe R Bolarinwa OA Saeed BQ Khuzwayo N Hlongwana KW, 2021. Knowledge, attitude, perception, and preventative practices towards COVID-19 in sub-Saharan Africa: a scoping review. PLoS One 16: e0249853. 33872330 10.1371/journal.pone.0249853 PMC8055009

[31] Zambia Statistics Agency , 2020. Zambia Demographic and Health Survey 2018. Lusaka, Zambia: Zambia Statistics Agency. Available at: www.DHSprogram.com. Accessed May 27, 2020.

[32] Santos REA da Silva MG do Monte Silva MC Barbosa DA do Vale Gomes AL Galindo LC da Silva Aragão R Ferraz-Pereira KN, 2021. Onset and duration of symptoms of loss of smell/taste in patients with COVID-19: a systematic review. Am J Otolaryngol 42: 102889. 33445036 10.1016/j.amjoto.2020.102889 PMC7833280

[33] Seytre B Barros C Bona P Fall B Konaté B Rodrigues A Varela O Yoro MB, 2021. Revisiting COVID-19 communication in western Africa: a health literacy-based approach to health communication. Am J Trop Med Hyg 105: 708–712. 34280141 10.4269/ajtmh.21-0013 PMC8592366

[34] Osuagwu UL , 2021. Misinformation about COVID-19 in sub-Saharan Africa: evidence from a cross-sectional survey. Health Secur 19: 44–56. 33606572 10.1089/hs.2020.0202 PMC9347271

[35] Chu L Fung HH Tse DCK Tsang VHL Zhang H Mai C, 2021. Obtaining information from different sources matters during the COVID-19 pandemic. Gerontologist 61: 187–195. 33388758 10.1093/geront/gnaa222 PMC7799117

[36] Mwananyanda L , 2021. Covid-19 deaths in Africa: prospective systematic postmortem surveillance study. BMJ 372: n334. 33597166 10.1136/bmj.n334 PMC7887952

[37] Gill CJ , 2022. Sustained high prevalence of COVID-19 deaths from a systematic post-mortem study in Lusaka, Zambia: one year later. *medRxiv*. doi:10.1101/2022.03.08.22272087.

[38] WHO , 2022. Water Sanitation and Health: Humanitarian Emergencies. Geneva, Switzerland: World Health Organization. Available at: https://www.who.int/teams/environment-climate-change-and-health/water-sanitation-and-health/environmental-health-in-emergencies/humanitarian-emergencies. Accessed August 24, 2022.

[39] Paludan-Müller A Boesen K Klerings I Jorgensen K Munkholm K, 2020. Hand cleaning with ash for reducing the spread of viral and bacterial infections: a rapid review. Cochrane Database Syst Rev 4: CD013597. 32343408 10.1002/14651858.CD013597 PMC7192094

[40] Adebisi YA Rabe A Lucero-Prisno DE, 2021. Risk communication and community engagement strategies for COVID-19 in 13 African countries. Health Promot Perspect 11: 137–147. 34195037 10.34172/hpp.2021.18 PMC8233683

[41] Juntunen A Kaiser JL Ngoma T Hamer DH Fink G Rockers PC Biemba G Scott NA, 2023. Lessons from a year of COVID-19 in Zambia: reported attendance and mask wearing at large gatherings in rural communities. Am J Trop Med Hyg 108: 384–393. 36509059 10.4269/ajtmh.22-0460 PMC9896318

[42] Republic of South Africa , 2021. Disaster Management Act: Regulations: Alert Level 3 during Coronavirus COVID-19 Lockdown. Pretoria, South Africa: South African Government. Available at: https://www.gov.za/covid-19/alert-level-3-coronavirus-covid-19-lockdown. Accessed July 30, 2022.

[43] Odour M, 2021. Covid-19: Ghanaians Issued Two Hours to Finalise Ceremonies, Funerals. Pointe-Noire, Republic of the Congo: Africanews. Available at: https://www.africanews.com/2021/07/26/covid-19-ghanaians-issued-two-hours-to-finalise-ceremonies-funerals/. Accessed July 30, 2022.

[44] Mbewa DO, 2021. Botswana Blames Surge in COVID-19 Cases on Funeral Gatherings. Nairobi, Kenya: CGTN Africa. Available at: https://africa.cgtn.com/2021/03/25/botswana-blames-surge-in-covid-19-cases-on-funeral-gatherings/. Accessed July 30, 2022.

[45] Mutsaka F, 2021. Zimbabwe Bans Traditional Funerals to Battle COVID-19 Spike. New York, NY: Associated Press. Available at: https://apnews.com/article/africa-zimbabwe-coronavirus-pandemic-5f0a3ae69bd5646d46801d4f2eea3885. Accessed July 30, 2022.

[46] Jaja IF Anyanwu MU Iwu Jaja CJ, 2020. Social distancing: how religion, culture and burial ceremony undermine the effort to curb COVID-19 in South Africa. Emerg Microbes Infect 9: 1077–1079. 32459603 10.1080/22221751.2020.1769501 PMC7336991

[47] Tiwari A , 2017. Assessing the impact of leveraging traditional leadership on access to sanitation in rural Zambia. Am J Trop Med Hyg 97: 1355–1361. 29016281 10.4269/ajtmh.16-0612 PMC5817733

[48] Haberer JE van der Straten A Safren SA Johnson MO Amico KR Del Rio C Andrasik M Wilson IB Simoni JM, 2021. Individual health behaviours to combat the COVID-19 pandemic: lessons from HIV socio-behavioural science. J Int AIDS Soc 24: e25771. 34339113 10.1002/jia2.25771 PMC8327691

